# Assessment of the Nutritional Value of Stems and Leaves of Australian Adzuki Bean

**DOI:** 10.3390/metabo13101062

**Published:** 2023-10-09

**Authors:** Joel B. Johnson, Ryan J. Batley, Pasmita Neupane, Surya P. Bhattarai, Tieneke Trotter, Diogo Fleury Azevedo Costa, Mani Naiker

**Affiliations:** School of Health, Medical and Applied Sciences, Central Queensland University, North Rockhampton, QLD 4701, Australia; ryan.batley@cqumail.com (R.J.B.); pasmita.neupane@cqumail.com (P.N.); s.bhattarai@cqu.edu.au (S.P.B.); t.trotter@cqu.edu.au (T.T.); d.costa@cqu.edu.au (D.F.A.C.); m.naiker@cqu.edu.au (M.N.)

**Keywords:** *Vigna angularis*, azuki bean, adzuki bean, phenolic acids, flavonoids

## Abstract

Adzuki bean has recently been proposed as a viable dual-purpose (grain-and-graze) crop for the Northern regions of Australia because of its successful use in semi-arid regions and its nitrogen fixation capacity to improve soil fertility and animal nutrition. However, there are very few studies on the phytochemical composition and nutritional value of the non-seed material. This study investigated the phenolic composition of the parts grown in the vegetative phase (leaves and stems) of nine Australian adzuki bean varieties for the first time. The total phenolic content (TPC) of the stem material (157–406 mg GAE/100 g) was 23–217% higher than that of commercial livestock feed, while the TPC of the leaf material (1158–1420 mg GAE/100 g) was 9–11 times higher. Using tandem liquid chromatography mass spectrometry (LC-MS), the major phenolic compounds identified were rutin, luteolin, salicylic acid, and quercetin-3-glucoside. The leaf and stem materials showed high levels of apparent in vitro dry matter digestibility, with no significant difference in total gas or methane production compared to lucerne hay. The results suggest that adzuki bean vegetative materials could be a high-value livestock fodder and support pursuing further in-depth studies into their nutritional value for livestock.

## 1. Introduction

Adzuki bean (*Vigna angularis*) is an annual pulse crop that originated in Eastern Asia and produces seeds with a dark red seed coat [1]. It is predominantly grown in Asian countries, notably Japan and China, where it has also been used in semi-arid regions [2]. The relatively high yields observed in drier environments justifies the growing interest in expanding adzuki bean cultivation in Australia, particularly as a legume rotation crop in the Northern regions [3]. This could open up opportunities for international market sales, with the adzuki bean market currently valued at over USD 184 million p.a. [4].

Recent work has highlighted the agronomic performance of various new adzuki bean cultivars that are suitable for growing under Australian conditions, with yields of 5 t/ha or more reported for several cultivars in controlled trials [5]. However, the significant agronomic inputs required, including irrigation, fertilisation, and disease control, often make commercial production challenging, resulting in a current annual production of less than 5000 tons in Australia [3].

One proposed solution to address the challenges of commercial adzuki bean production in Australia is by adopting a dual-purpose approach known as the “grain and graze” cropping system [5]. In this system, the adzuki bean crop serves both as livestock fodder and for seed harvest [6,7]. Typically, the crop is grazed during the vegetative stage and then allowed to regrow before being harvested at maturity, although the reverse approach of harvesting the seed at maturity and then allowing livestock to graze the residual stubble can also be considered. Despite adzuki bean being reported as a suitable livestock fodder [1], there is limited research on its nutritional quality and suitability for this purpose, warranting further studies.

In addition to providing basic nutritional value, adzuki bean vegetative material has been found to contain high levels of phenolic and antioxidant compounds. Phenolic compounds are known to be synthesised in response to environmental stress, which is not uncommon in the typically dry Northern Australian regions. Some previous studies have indicated that incorporating higher amounts of these compounds into livestock diets can provide a range of benefits, such as enhanced growth rates, improved meat/milk quality, increased utilisation of other nutrients in the fodder, and reduced methane emissions [8,9,10].

However, there is currently limited research on the phytochemical content and phenolic composition of the vegetative material of adzuki bean. Hence, the present study aimed to investigate the chemical composition and assess the nutritional value of the vegetative material from nine different adzuki bean genotypes grown in Australia. We build on our earlier research on these genotypes [5] to investigate their phytochemical composition and potential suitability as livestock fodder. This research seeks to bridge the knowledge gap and provide valuable insights into the potential use of adzuki beans as a beneficial feed source for livestock.

## 2. Materials and Methods

### 2.1. Vegetative Material and Preparation

Nine Adzuki bean genotypes sourced from AgriVentis Technologies Ltd. (https://www.agriventistechnologies.com.au/) (accessed on 19 February 2023) were cultivated in a controlled raised bed field trial in Rockhampton, Queensland, following the methods as described in Johnson, et al. [5]. Upon reaching physiological maturity, which occurred approximately 88–94 days after sowing, the crop was manually harvested. The vegetative material was separated into leaves and stems before being oven-dried at 60 °C until constant weight and ground to a fine powder (Sunbeam Multi grinder II EMO405).

### 2.2. Measurement of Proximate Chemical Composition

The protein content of the dried samples was previously determined and reported in Johnson, et al. [5], using a LECO TruMac Series Carbon and Nitrogen Analyser (Saint Joseph, MI, USA), applying a 6.25× conversion factor. Only a single replicate was performed on each sample, as our laboratory’s typical reproducibility of replicate samples using this method is 0.10% *w*/*w*.

Similarly, the gross energy content was measured using a bomb calorimeter (Changsha Kaiyuan Instruments Co. 5E-C5500), and has been reported in Johnson, et al. [5]. Again, a single replicate was conducted for each sample; the typical reproducibility for gross energy analysis in our laboratory is 0.02 MJ/kg.

### 2.3. Extraction of Phenolics and Measurement of TPC

The polyphenols and other polar phytochemicals were extracted from the stem and leaf material using 90% *v*/*v* methanol, as described previously [5]. The extractions were performed in duplicate. The total phenolic content (TPC) was determined using the Folin–Ciocalteau method, as described in [5], with results expressed as gallic acid equivalents (GAE) per 100 g of sample (dry matter basis). The TPC data were briefly reported in Johnson, et al. [5], but is included here for completeness and ease of comparison to the targeted phenolic profiling data.

### 2.4. Targeted Phenolic Profiling Using LC-MS/MS

The targeted quantification of 31 polyphenol compounds was performed using a Shimadzu LCMS-8040 tandem mass spectrometry system (LC-MS/MS), following the methods described by Johnson, et al. [5] for adzuki bean seeds. The LC-MS/MS data were collected and analysed using the LabSolutions software (Shimadzu, Kyoto, Japan).

### 2.5. Screening for Acetylcholinesterase Inhibition Activity

In accordance with our previous findings of moderately strong inhibitory activity against acetylcholinesterase (AChE) in adzuki bean seedcoat material [11], screening for AChE inhibition was performed on the stem and leaf extracts from one of the adzuki bean genotypes (AVTAB#1). The screening was conducted following methods adapted from Jo, et al. [12], which involved combining 40 µL of extract with 160 µL of 0.2 M phosphate buffer, 80 µL of 1 mM DTNB, 10 µL of 2 U/mL acetylcholinesterase, and 15 µL of 8 mM acetylthiocholine iodide. The absorbance at 405 nm, measured using a microplate reader (Bio-Rad iMark), was used to monitor the amount of substrate processed and thus determine the inhibitory activity of the extracts. However, due to the lack of activity observed in both samples, AChE screening was not conducted for the remaining genotypes.

### 2.6. Apparent In Vitro Dry Matter Digestibility

The prepared leaf and stem material from selected adzuki bean genotypes (chosen based on their protein contents and other compositional characteristics) was subjected to apparent in vitro digestibility testing to determine whether these vegetative parts would be suitable as ruminant fodder. A lucerne hay (*Medicago sativa*) was used as a control for comparison. Fermentations were conducted in duplicate, with individual replicates contained in a 250 mL reaction vessel. Approximately 0.5 g of plant material was weighed into individual Ankom F-57 filter bags (Ankom Technology, Macedon, NY, USA), which had previously been treated with acetone, dried, and weighed as per the manufacturer’s instructions. Bags were added to the reaction vessel, along with 100 mL of pre-warmed buffer [13], before being placed into a shaking water bath set to 39 °C to equilibrate for thirty minutes. A reducing agent was added (2 mL) to remove oxygen from the buffer prior to the addition of 25 mL of well-mixed rumen fluid. The vessels were flushed with CO_2_ before being topped with Ankom RF gas production modules (Ankom Technology, Macedon, NY, USA), and then flushed with CO_2_ an additional three times as per the manufacturer’s instructions. The fermentations were allowed to run for 48 h with the shaking of the water bath set to 50 RPM, and upon completion, the reaction vessels were placed in an ice bath to stop fermentation. The individual filter bags were collected into a beaker containing ice water before being flushed and squeezed in cold running water until it ran clear. Filter bags were placed in an oven set to 105 °C for 24 h before being allowed to cool to room temperature in the presence of a desiccant and then re-weighed. Apparent in vitro dry matter digestibility was determined as the difference in the pre- and post-fermentation plant material mass, with an allowance made for absorbance of rumen fluid into the filter bag using blanks.

### 2.7. Rumen Fluid Collection

Cattle rumen fluid was collected post-slaughter from an AUS-MEAT A+ accredited commercial abattoir (JBS Australia, Rockhampton, QLD, Australia) as described previously [14]. The fluid collected was an aggregate of samples from 4 Brahman (Bos taurus indicus) cross steers (live weight 241 ± 30 kg), reared on a free-forage diet consisting primarily of Buffel grass (*Cenchrus ciliaris* L.) and Green Panic (*Panicum maximum* var. trichoglume).

### 2.8. Total Gas Production

The Ankom RF gas production modules were utilised to determine the total gas produced during the fermentation. Parameters were set to maintain a maximum pressure of 3 psi, with gas pressure measured every 60 s and cumulative pressure recorded every 20 min. Upon exceeding the maximum pressure, the vessels would vent for 250 ms, adding to the cumulative pressure of the fermentation. The natural gas law was applied to the total cumulative pressure at the end of the fermentation to determine the total gas produced, which was reported as mL/g of fermented substrate.

### 2.9. Total Methane Production

At the end of the fermentation and prior to being added to the ice bath, 20 mL of gas was sampled from the headspace with a gas-tight syringe from a side port on the reaction vessel. This sample was transferred to a 12 mL Exetainer vial (Labco, Lampeter, Ceredigion) for further analysis. Gas chromatography was used to measure methane concentration from the subsampled headspace on an Agilent 6890N GC (Santa Clara, CA, USA) fitted with a Supelco (Bellefonte, PA, USA) 80/100 HayeSep Q 3FT × 1/8 IN × 2.1 mm stainless steel column and a flame ionisation detector. Helium was used as the carrier gas at a flow rate of 11.1 mL/min; the injection temperature was 100 °C; column temperature was 65 °C held for 2 min; detector temperature was 250 °C; and the injection volume was 250 µL. Results of total methane were reported as mL/g of fermented substrate.

### 2.10. Amylase Neutral Detergent Fibre

Amylase neutral detergent fibre was measured using an Ankom (Macedon, NY, USA) model 200 fibre analyser as per the manufacturer’s instructions, with results reported as a percentage of the raw substrate.

### 2.11. Ammonia

Ammonia was calculated using a modified version of the colorimetric assay established by Baethgen and Alley [15]. An 8 mL sample of the in vitro fluid was taken from the reaction vessel after fermentation in the ice bath and added to 2 mL of 0.5 M H_2_SO_4_ to stabilise the ammonium before being stored at −20 °C prior to further analysis. The thawed fluid was centrifuged (2400× *g*, 20 min) and 100 µL of supernatant was diluted into 9.9 mL of distilled water. An aliquot of 800 µL of diluted sample, 1200 µL of tartrate buffer, 800 µL of sodium salicylate/sodium nitroprusside solution, and 400 µL of dilute sodium hypochlorite were combined in a 10 mL tube, vortexed, and incubated for 15 min at 37 °C prior to having absorbance measured on a Genysys 10 s UV-vis spectrophotometer (Thermo Scientific, Waltham, MA, USA) set to 660 nm. The resulting concentrations were calculated using a calibration curve of N NH_4_ standards prior to conversion to N NH_3_ by multiplying by 0.9685 (molar mass of N NH_3_/molar mass of N NH_4_). Results were reported in mg N NH_3_/L, after the subtraction of the average ammonia measured in the blank samples to account for ammonia introduced by the rumen fluid.

### 2.12. Volatile Fatty Acids

Gas chromatography-mass spectrometry (GC-MS) was used to measure volatile fatty acids (VFAs). A 4 mL sample of fluid was taken from the reaction vessel after fermentation while in the ice bath and added to 1 mL of 20% metaphosphoric acid that had previously been spiked to 11 mM 4-methylvaleric acid before being stored at −20 °C prior to further analysis. The thawed fluid was centrifuged (1000× *g*, 10 min), and approximately 1.5 mL of the supernatant was passed through a 0.22 µm syringe filter into a sample vial. Multi-acid standards were also prepared, with 1 mL filtered into a sample vial along with 200 µL of the metaphosphoric/4-methylvaleric spiking mix to maintain the same ratio as the samples. Samples were analysed on a Shimadzu (Nakagyo-ku, Kyoto, Japan) QP2010 Plus System fitted with an autoinjector/autosampler (AOC-20i/s) and an Agilent (Santa Clara, CA, USA) HP-INNOWax column (30 m × 0.25 mm × 0.25 µm). The injection temperature was 250 °C, the ion source temperature was 230 °C, and helium was used as the carrier gas at a column flow rate of 1.27 mL/min. The column temperature started at 80 °C held for 2 min, ramped to 230 °C at 10 °C per minute, then held for 5 min. A split injection mode was used at a ratio of 30 and an injection volume of 0.2 µL. Scanning between 30 and 450 *m*/*z* was used as the acquisition mode. Three quality control multi-acid standards (low, medium, and high) were used to validate the GC-MS method, with recoveries of 102.6 ± 0.9%. Results were reported in mM after the subtraction of average VFAs measured in the blank samples to account for those introduced by the rumen fluid. Values for butyric and valeric isomers were combined for ease of reporting.

### 2.13. Data Analysis

R Studio, running R 4.0.5 [16], was used to conduct the statistical analyses. Where applicable, results are presented as the mean ± 1 standard deviation.

## 3. Results and Discussion

### 3.1. Proximate Nutritional Composition

Proximate compositional analysis of the adzuki bean vegetative material has been reported in our previous publication [5], but is reproduced here for completeness (Table 1). The protein content in the stem samples ranged from 7.4 to 12.8%, while it was higher in all of the leaf samples, ranging from 19.5 to 24.0%. These protein levels were comparable to the mean protein content reported by Fulkerson, et al. [17] in the leaves of Australian-grown legumes during the summer season and higher than most alternative forage crops in another study [18].

Similarly, the gross energy content of the leaf samples was reasonably high, ranging from 15.8 to 18.0 MJ/kg. This is comparable to that of native pasture grasses from Queensland, Australia (15.5–17.8 MJ/kg) [19] and alpine forage crops in an international study (17.2–18.9 MJ/kg) [20]. The gross energy content of the stem samples was slightly lower (14.5–16.0 MJ/kg) but still within a reasonable range. It appears that vegetative portions of the adzuki bean plant (combined stem and leaves) could provide adequate nutritional intake for livestock fodder in terms of gross energy and protein. However, the fibre fraction affects both feed intake and animal performance; thus, the apparent digestibility was evaluated in vitro, with those results presented later in this work (Section 3.5).

### 3.2. Total Phenolic Contents

**Figure 1:**
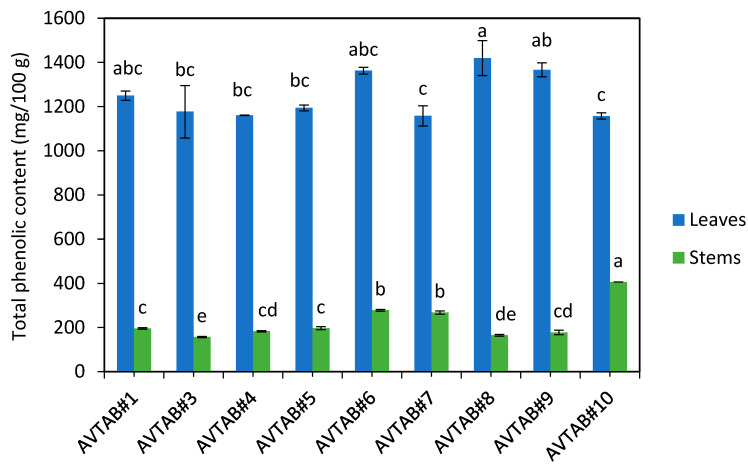
Total phenolic content (TPC) of adzuki bean leaf and stem material. The graph is drawn from data previously reported in Johnson, et al. [5]. The letters above each bar represent the statistical differences based on one-way ANOVAs followed by post hoc Tukey testing at α = 0.05. Note that statistical analyses of the leaf and stem samples were conducted separately, so they are not directly comparable.

As illustrated in Figure 1, the total phenolic content of the leaf material from adzuki bean varieties (1158–1420 mg GAE/100 g) was significantly higher compared to the stem material (157–406 mg GAE/100 g). To provide context, our laboratory’s testing has shown that the TPC of typical commercial livestock feed, consisting of 90% barley and 10% hay, is approximately 128 mg GAE/100 g. This indicates that the stem material from all adzuki bean varieties had a higher TPC than the commercial livestock feed. Furthermore, the adzuki bean leaf material, which constitutes a substantial portion of the entire vegetative matter, contained around 10 times higher TPC compared to the commercial diet. This suggests that adzuki bean vegetative material could be used as a high-value livestock fodder with potential benefits such as improved nutrient utilisation and growth rates. Our experimental incubations in rumen fluid are the first step to assessing the nutritional value of adzuki bean (Section 3.5). However, further research would be required to establish the extent of these benefits through feeding trials.

### 3.3. Phenolic Profiling by LC-MS/MS

Using the targeted phenolic profiling method, 21 compounds were confirmed in the leaves (Table 2) and 14 compounds in the stems (Table 3).

Among the leaves, rutin was the most abundant compound, followed by luteolin, quercetin-3-glucoside, and salicylic acid. In the stems, rutin was again the most abundant compound, although at much lower concentrations compared to the leaves (3.2–97.1 mg/100 g, compared to 167.2–407.7 mg/100 g for the leaves). Other major compounds included salicylic acid and luteolin.

**Figure 2:**
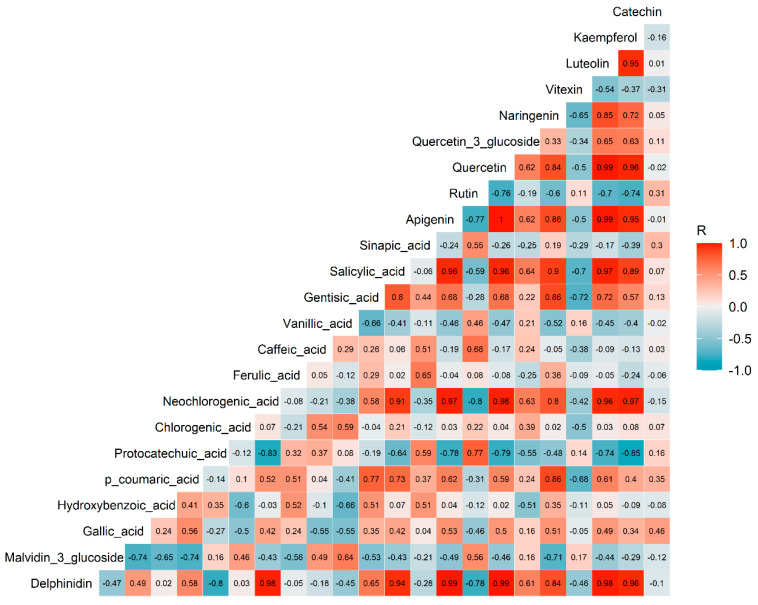
Correlation plot of the phenolics in adzuki bean leaves. The values in each square represent the Pearson R correlation coefficients. Correlation coefficients above 0.47 or below −0.47 were considered statistically significant at α = 0.05.

As depicted in Figure 2, there were positive correlations observed among several phenolic compounds, particularly among a group of flavonoids including kaempferol, luteolin, naringenin, quercetin, and apigenin. Neochlorogenic acid also exhibited positive correlations with some of these flavonoids.

**Figure 3:**
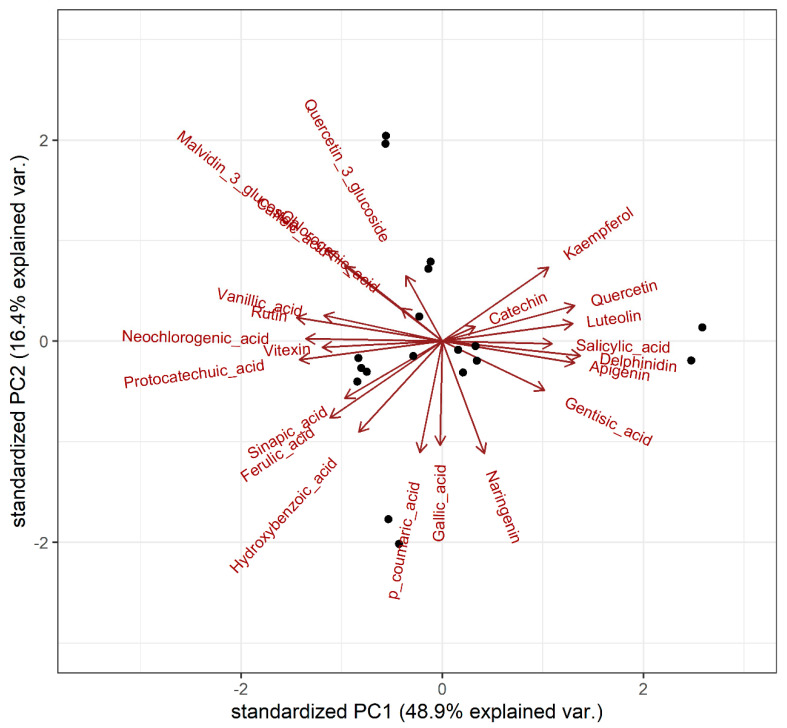
Scores plot of principal component analysis for leaf phenolic contents, based on the centred log-ratio transformed data.

This was supported by principal component analysis (PCA) performed on the centred log-ratio transformed data (Figure 3). A cluster of compounds dominated by flavonoids was observed on the right side of the score plot, spanning across the first two principle components that explained 65% of the phenolic variance (see Figure 3). This cluster included kaempferol, catechin, quercetin, luteolin, and apigenin, along with delphinidin, salicylic acid, and gentisic acid. These findings further confirm that among the samples tested, varieties with high levels of one flavonoid tended to exhibit high levels of the other flavonoids (but not necessarily high levels of phenolic acids), and vice versa. This could potentially be attributed to the up- or down-regulation of genes involved in the high-level control of flavonoid synthesis. The same degree of clustering between flavonoids was not observed in the stem samples.

### 3.4. Acetylcholinesterase Inhibitory Activity

At the highest tested concentration of the methanol extracts from the stem and leaves (36 mg/mL) of the tested genotype (AVTAB#1), only weak inhibitory activity against AChE was observed. The inhibition was 36% for the leaf sample and 39% for the stem sample. In contrast, our previous study reported 89% inhibition in the seedcoat extract at the same concentration [11]. Consequently, the AChE inhibitory activity was not measured for the stem and leaf extracts of the other adzuki bean genotypes.

### 3.5. In Vitro Digestibility

Finally, the in vitro dry matter digestibility (IVDMD) of selected adzuki bean leaf and stem samples was investigated to evaluate their potential for use as livestock fodder. Despite not fully replicating the complexities of in vivo digestion, the information gathered in vitro provides valuable insights into the potential nutrient availability of feeds in the rumen. This is particularly important for demonstrating if adzuki bean is suitable for use as a dual-purpose crop.

Compared to a standard livestock fodder (lucerne hay), the adzuki bean leaf material from all genotypes tested did not show any significant difference in IVDMD or in neutral detergent fibre content (NDF) (Table 4). The NDF is a critical component of the diet of ruminants and plays a significant role in their nutrition. Unlike monogastric animals, ruminants can break down fibre through fermentation because of a symbiotic relationship with the various microorganisms in their forestomach. This is of great importance when considering a dual-purpose crop for Northern Australia, from which about 70% of the red meat in the country is produced in extensive grazing systems. Legumes such as adzuki bean and lucerne hay, used in the comparison, have less total NDF and lower NDF digestibility compared to grasses [21]. Highly digestible fibre stays in the rumen for a shorter time, which promotes greater dry matter intake. Despite being advantageous for high-yield herds focused on maximising forage consumption, for beef cattle in extensive grazing systems, the simple availability of fodder of some sort may come first in importance. As observed here, the introduction of adzuki bean into Northern systems would not only generate and make more forage available but also be a high-quality material. The latter statement can be backed by the observation that there were no significant differences in the amount of total gas, ammonia, or methane produced amongst the forages in the assay when compared to lucerne.

Despite the promising results, some genotypes (particularly AVTAB#6) produced significantly less of the volatile fatty acids (VFAs) acetic acid and propionic acid but not butyric or valeric acid. Along with butyrate, both acetate and propionate represent the main sources of energy used by a ruminant. While butyrate is mainly used to provide energy for their digestive system, specifically within the rumen, acetate and propionate are the key drivers of animal performance. This indicates that these genotypes may provide slightly less energy to the host animal compared to lucerne hay. However, as indicated above, the significance of quality may vary depending on specific factors. Being available in sufficient amounts in a given environment may play a pivotal role, particularly in the seasonally dry tropical regions of the country. The adzuki bean’s life cycle, from seeding to harvest, was found to be significantly faster (i.e., 69 days vs. 89 days and above) when compared to five other types of beans [22]. This might be essential for their survival and adaptation to such challenging conditions as the ones found in Northern Australia. A faster cycle means that adzuki beans have reduced overall water requirements, and this would literally increase the chances of survival in such variable environments. In the current study, manual harvest occurred approximately 88–94 days after sowing.

On the other hand, a range of differences were seen between the in vitro digestibility of the selected adzuki bean stem samples and lucerne hay. In general, the IVDMD of adzuki bean stems was not different from that of lucerne but was found to have a higher NDF content (Table 5). The stems also tended to produce less ammonia, but all samples except AVTAB#6 were above 20 mg of NH_3_-N/L of rumen fluid, as suggested by Satter and Slyter [23] as the limiting concentration of N required in the rumen for efficient microbial synthesis. Despite being efficient at recycling N in low-protein diets [24], the addition of this valuable nutrient is one of the great advantages of introducing legumes in Northern production systems. The quality of this plant part still had similar levels of total gas and methane production when compared to lucerne, indicating that it was material of good quality for ruminants that could potentially utilise this dual-purpose crop. Finally, some genotypes (particularly AVTAB#6 and AVTAB#9) produced significantly lower amounts of acetic, propionic, and/or valeric acid. As indicated before, the first three VFAs are the main energy sources for ruminants, but the lack of differences in IVDMD and total gas production suggests one should expect no differences in the efficiency of the use of these forage materials by rumen microbes. In contrast, valeric acid is one of the branched-chain VFAs, which are required by cellulolytic bacteria to utilise ammonia when they synthesise their own proteins [25]. However, as already stated, the lack of differences in IVDMD and gas production suggests that these were present in sufficient amounts.

## 4. Conclusions

This study investigated the phytochemical composition and assessed in vitro the nutritional value of adzuki bean leaves and stems across various Australian adzuki bean genotypes. The TPCs were significantly higher in leaves compared to stems, with leaves showing almost an order of magnitude higher TPCs. However, the TPCs of the stem materials were still higher than those of a commercial livestock feed, suggesting that consumption of adzuki bean vegetative material could potentially lead to better growth and health outcomes in livestock. A total of 21 phenolics were identified in the leaf samples and 14 in the stem samples, with chemometric data analysis demonstrating the correlation/clustering of most flavonoids found in the leaves. The samples did not show any inhibitory activity against acetylcholinesterase, unlike the positive results previously observed for adzuki bean seedcoat extracts. The assessment of the nutritional value of both the leaf and stem material demonstrated a generally high in vitro dry matter digestibility and neutral detergent fibre, resulting in no significant differences in methane or total gas production when compared to lucerne hay. This suggests both plant parts could be used as fodder or are relatively high-quality, despite the fact that some of the genotypes produced moderately lower levels of certain volatile fatty acids, particularly for the stem substrates. The latter results indicate that these samples could provide slightly less available energy to livestock when compared to lucerne hay, but certainly would represent a very interesting option of fodder for beef cattle production systems in Northern Australia. One possible challenge to expanding the Australian adzuki bean industry is the limited domestic market for the seed material; this should be considered by future researchers or potential growers. However, the high antioxidant and phenolic content of the seed material—along with the associated potential health benefits—may make this crop more favourable for domestic, high-value uses.

## Figures and Tables

**Table 1:** Indicative protein and gross energy contents of the adzuki bean vegetative material, reproduced from Johnson, et al. [5]. Values are from a single determination (typical reproducibility is 0.1% *w*/*w* for protein and 0.02 MJ/kg for gross energy).

Variety	Protein Content (%)		Gross Energy Content (MJ/kg)	
	Leaves	Stems	Leaves	Stems
AVTAB#1	19.5	7.4	17.0	14.5
AVTAB#3	20.8	7.4	17.6	16.1
AVTAB#4	20.4	8.1	15.8	14.8
AVTAB#5	21.7	7.4	18.0	15.3
AVTAB#6	22.3	8.9	18.0	15.6
AVTAB#7	21.0	7.5	16.9	16.0
AVTAB#8	20.2	7.9	17.9	16.0
AVTAB#9	24.0	8.9	17.7	16.0
AVTAB#10	22.6	12.8	16.0	15.4

**Table 2:** Phenolic content of the adzuki bean leaves presented as mg/100 g (dry-weight basis). Entries in the same row with different superscript letters indicate significant differences based on a one-way ANOVA followed by post hoc Tukey testing at α = 0.05.

Compound	AVTAB#1	AVTAB#3	AVTAB#4	AVTAB#5	AVTAB#6	AVTAB#7	AVTAB#8	AVTAB#9	AVTAB#10	ANOVA
Gallic acid	0.05 ± 0.00 ^c^	0.16 ± 0.00 ^ab^	0.13 ± 0.00 ^b^	0.14 ± 0.01 ^b^	0.15 ± 0.03 ^ab^	0.19 ± 0.02 ^a^	0.11 ± 0.01 ^b^	0.19 ± 0.00 ^a^	0.14 ± 0.01 ^b^	<0.001
Protocatechuic acid	0.78 ± 0.06 ^ab^	0.95 ± 0.08 ^a^	0.69 ± 0.01 ^b^	0.80 ± 0.05 ^ab^	0.80 ± 0.06 ^ab^	0.42 ± 0.07 ^c^	0.97 ± 0.03 ^a^	0.86 ± 0.03 ^ab^	0.84 ± 0.04 ^ab^	<0.001
Gentisic acid	0.25 ± 0.13 ^d^	0.47 ± 0.04 ^c^	0.25 ± 0.01 ^cd^	0.18 ± 0.04 ^d^	0.25 ± 0.01 ^cd^	1.29 ± 0.05 ^a^	1.30 ± 0.04 ^a^	0.81 ± 0.09 ^b^	0.41 ± 0.00 ^cd^	<0.001
4-hydroxybenzoic acid	0.83 ± 0.02 ^c^	1.35 ± 0.25 ^ab^	1.18 ± 0.01 ^bc^	1.34 ± 0.05 ^ab^	1.01 ± 0.13 ^bc^	1.24 ± 0.04 ^bc^	1.67 ± 0.04 ^a^	1.24 ± 0.08 ^bc^	1.42 ± 0.10 ^ab^	0.001
Neochlorogenic acid	ND	ND	ND	ND	ND	0.29 ± 0.07	ND	ND	ND	-
(+)-Catechin	ND	ND	ND	ND	0.05 ± 0.01	ND	ND	0.27 ± 0.01	ND	<0.001
Caffeic acid	0.91 ± 0.01 ^a^	0.64 ± 0.01 ^b^	0.47 ± 0.05 ^cd^	0.46 ± 0.01 ^cd^	0.72 ± 0.05 ^b^	0.52 ± 0.01 ^c^	0.95 ± 0.03 ^a^	0.63 ± 0.02 ^b^	0.40 ± 0.01 ^d^	<0.001
Chlorogenic acid	0.32 ± 0.02 ^a^	0.09 ± 0.00 ^d^	0.09 ± 0.00 ^d^	0.08 ± 0.01 ^d^	0.21 ± 0.01 ^b^	0.17 ± 0.02 ^bc^	0.15 ± 0.01 ^c^	0.16 ± 0.01 ^bc^	0.18 ± 0.01 ^bc^	<0.001
Cyanidin 3-glucoside	ND	ND	ND	ND	ND	ND	ND	ND	ND	-
Salicylic acid	3.03 ± 0.00 ^c^	2.49 ± 0.12 ^d^	1.79 ± 0.04 ^e^	1.90 ± 0.03 ^e^	2.67 ± 0.10 ^d^	8.24 ± 0.02 ^a^	3.96 ± 0.02 ^b^	3.87 ± 0.03 ^b^	2.46 ± 0.05 ^d^	<0.001
Vanillic acid	0.79 ± 0.03 ^a^	0.29 ± 0.01 ^cd^	0.37 ± 0.04 ^cd^	0.59 ± 0.07 ^b^	0.80 ± 0.09 ^a^	0.25 ± 0.03 ^d^	0.30 ± 0.00 ^cd^	0.39 ± 0.04 ^cd^	0.43 ± 0.02 ^bc^	<0.001
Syringic acid	ND	ND	ND	ND	ND	ND	ND	ND	ND	-
p-Coumaric acid	1.26 ± 0.07 ^de^	1.57 ± 0.20 ^cd^	1.11 ± 0.02 ^e^	1.31 ± 0.03 ^de^	1.79 ± 0.15 ^bc^	2.29 ± 0.08 ^a^	2.00 ± 0.00 ^ab^	2.06 ± 0.07 ^ab^	1.90 ± 0.09 ^abc^	<0.001
Malvidin 3-glucoside	0.04 ± 0.00 ^a^	0.01 ± 0.00 ^bc^	0.02 ± 0.00 ^b^	0.02 ± 0.00 ^b^	0.01 ± 0.00 ^b^	<0.01 ± 0.00 ^d^	0.01 ± 0.00 ^bcd^	0.01 ± 0.00 ^bcd^	<0.01 ± 0.00 ^cd^	<0.001
Ferulic acid	1.27 ± 0.08 ^d^	1.71 ± 0.19 ^ab^	1.35 ± 0.01 ^cd^	1.66 ± 0.09 ^abc^	1.93 ± 0.00 ^a^	1.61 ± 0.08 ^abcd^	1.96 ± 0.11 ^a^	1.56 ± 0.05 ^bcd^	1.86 ± 0.02 ^ab^	<0.001
Vitexin	0.06 ± 0.02 ^cd^	0.21 ± 0.02 ^b^	0.28 ± 0.02 ^a^	0.19 ± 0.01 ^b^	0.19 ± 0.02 ^b^	0.01 ± 0.00 ^d^	0.03 ± 0.00 ^d^	0.03 ± 0.00 ^d^	0.11 ± 0.01 ^c^	<0.001
Rutin	406.35 ± 4.59 ^a^	370.10 ± 6.47 ^b^	316.03 ± 0.94 ^c^	323.92 ± 1.90 ^c^	401.81 ± 3.70 ^a^	167.22 ± 0.83 ^e^	407.74 ± 17.65 ^a^	394.20 ± 6.75 ^ab^	278.44 ± 9.40 ^d^	<0.001
Quercetin 3-glucoside	4.26 ± 0.20 ^b^	3.66 ± 0.07 ^cd^	2.64 ± 0.08 ^ef^	3.43 ± 0.10 ^d^	4.02 ± 0.28 ^bc^	4.99 ± 0.19 ^a^	2.79 ± 0.05 ^e^	3.66 ± 0.04 ^cd^	2.20 ± 0.01 ^f^	<0.001
Sinapic acid	0.30 ± 0.05 ^c^	0.46 ± 0.14 ^bc^	0.28 ± 0.03 ^c^	0.44 ± 0.03 ^bc^	0.59 ± 0.00 ^bc^	0.28 ± 0.04 ^c^	0.91 ± 0.11 ^a^	0.63 ± 0.09 ^ab^	0.41 ± 0.10 ^bc^	<0.001
Ellagic acid	ND	ND	ND	ND	ND	ND	ND	ND	ND	-
Phloridzin	ND	ND	ND	ND	ND	ND	ND	ND	ND	-
Myricetin	ND	ND	ND	ND	ND	ND	ND	ND	ND	-
Resveratrol	ND	ND	ND	ND	ND	ND	ND	ND	ND	-
Pelargonidin	ND	ND	ND	ND	ND	ND	ND	ND	ND	-
Delphinidin	0.02 ± 0.01 ^c^	0.12 ± 0.05 ^bc^	0.11 ± 0.01 ^bc^	0.01 ± 0.00 ^c^	0.05 ± 0.01 ^c^	2.39 ± 0.01 ^a^	0.21 ± 0.03 ^b^	0.22 ± 0.08 ^b^	0.04 ± 0.02 ^c^	<0.001
Quercetin	0.07 ± 0.02 ^cd^	0.12 ± 0.00 ^cd^	0.15 ± 0.02 ^cd^	0.01 ± 0.00 ^d^	0.02 ± 0.01 ^d^	2.42 ± 0.17 ^a^	0.27 ± 0.00 ^bc^	0.41 ± 0.00 ^b^	0.02 ± 0.00 ^d^	<0.001
Luteolin	3.22 ± 0.02 ^c^	3.34 ± 0.24 ^c^	3.01 ± 0.03 ^c^	3.51 ± 0.02 ^c^	2.99 ± 0.06 ^c^	14.00 ± 0.26 ^a^	4.79 ± 0.23 ^b^	5.07 ± 0.08 ^b^	2.23 ± 0.10 ^d^	<0.001
Cyanidin	ND	ND	ND	ND	ND	ND	ND	ND	ND	-
Kaempferol	0.63 ± 0.09 ^bc^	0.50 ± 0.01 ^bc^	0.75 ± 0.01 ^b^	0.38 ± 0.04 ^bc^	0.37 ± 0.07 ^bc^	2.26 ± 0.30 ^a^	0.54 ± 0.02 ^bc^	0.48 ± 0.06 ^bc^	0.27 ± 0.09 ^c^	<0.001
Naringenin	0.16 ± 0.00 ^e^	0.19 ± 0.00 ^de^	0.17 ± 0.01 ^e^	0.18 ± 0.00 ^de^	0.22 ± 0.00 ^cd^	0.39 ± 0.02 ^a^	0.29 ± 0.02 ^b^	0.25 ± 0.00 ^c^	0.25 ± 0.01 ^c^	<0.001
Apigenin	0.54 ± 0.02 ^d^	0.65 ± 0.02 ^cd^	0.61 ± 0.01 ^d^	0.63 ± 0.01 ^d^	0.60 ± 0.01 ^d^	3.07 ± 0.11 ^a^	0.79 ± 0.02 ^c^	1.00 ± 0.03 ^b^	0.60 ± 0.01 ^d^	<0.001
Sum of identified compounds	425.2 ± 4.8 ^a^	389.1 ± 6.5 ^b^	331.5 ± 1.1 ^c^	341.2 ± 1.8 ^c^	421.3 ± 2.8 ^a^	213.5 ± 1.7 ^e^	431.7 ± 17.4 ^a^	418.0 ± 6.9 ^ab^	294.6 ± 10.0 ^d^	<0.001

ND = no data.

**Table 3:** Phenolic content of the adzuki bean stems reported as mg/100 g (dry-weight basis). Entries in the same row with different superscript letters indicate significant differences based on a one-way ANOVA followed by post hoc Tukey testing at α = 0.05.

Compound	AVTAB#1	AVTAB#3	AVTAB#4	AVTAB#5	AVTAB#6	AVTAB#7	AVTAB#8	AVTAB#9	AVTAB#10	ANOVA
Gallic acid	0.05 ± 0.00 ^d^	0.06 ± 0.01 ^cd^	0.05 ± 0.00 ^d^	0.08 ± 0.01 ^bcd^	0.10 ± 0.00 ^b^	0.10 ± 0.02 ^bc^	0.07 ± 0.01 ^bcd^	0.15 ± 0.01 ^a^	0.10 ± 0.00 ^b^	<0.001
Protocatechuic acid	0.15 ± 0.00 ^cd^	0.11 ± 0.01 ^d^	0.12 ± 0.01 ^cd^	0.13 ± 0.00 ^cd^	0.19 ± 0.02 ^bc^	0.68 ± 0.01 ^a^	0.12 ± 0.01 ^cd^	0.23 ± 0.04 ^b^	0.14 ± 0.02 ^cd^	<0.001
Gentisic acid	0.07 ± 0.02 ^d^	0.12 ± 0.02 ^cd^	0.18 ± 0.03 ^bc^	0.17 ± 0.01 ^bc^	0.23 ± 0.01 ^b^	1.34 ± 0.03 ^a^	0.13 ± 0.03 ^cd^	0.14 ± 0.01 ^cd^	0.21 ± 0.00 ^b^	<0.001
4-hydroxybenzoic acid	ND	ND	ND	ND	ND	ND	ND	ND	ND	-
Neochlorogenic acid	ND	ND	ND	ND	ND	ND	ND	ND	ND	-
(+)-Catechin	ND	ND	ND	ND	0.26 ± 0.03	ND	ND	ND	ND	-
Caffeic acid	ND	ND	ND	ND	ND	ND	ND	ND	ND	-
Chlorogenic acid	ND	ND	ND	ND	0.06 ± 0.01	0.02 ± 0.00	ND	ND	ND	-
Cyanidin 3-glucoside	ND	ND	ND	ND	ND	ND	ND	ND	ND	-
Salicylic acid	4.91 ± 0.01 ^d^	7.61 ± 0.28 ^b^	3.63 ± 0.02 ^e^	3.33 ± 0.10 ^e^	4.80 ± 0.12 ^d^	13.25 ± 0.42 ^a^	8.30 ± 0.01 ^b^	3.98 ± 0.13 ^e^	6.35 ± 0.09 ^c^	<0.001
Vanillic acid	0.09 ± 0.01 ^ab^	0.07 ± 0.01 ^bc^	0.10 ± 0.01 ^ab^	0.13 ± 0.02 ^a^	0.09 ± 0.00 ^ab^	0.04 ± 0.02 ^cd^	0.02 ± 0.00 ^d^	0.03 ± 0.01 ^cd^	0.03 ± 0.01 ^cd^	<0.001
Syringic acid	ND	ND	ND	ND	ND	ND	ND	ND	ND	-
p-Coumaric acid	0.45 ± 0.02 ^b^	0.51 ± 0.01 ^b^	0.42 ± 0.04 ^bc^	0.21 ± 0.08 ^d^	0.30 ± 0.00 ^cd^	0.67 ± 0.05 ^a^	0.40 ± 0.04 ^bc^	0.22 ± 0.01 ^d^	0.29 ± 0.01 ^cd^	<0.001
Malvidin 3-glucoside	ND	ND	ND	ND	ND	ND	ND	ND	ND	-
Ferulic acid	ND	ND	ND	ND	ND	ND	ND	ND	ND	-
Vitexin	ND	ND	ND	ND	ND	ND	ND	ND	ND	-
Rutin	10.16 ± 0.18 ^d^	16.71 ± 0.10 ^c^	33.50 ± 1.71 ^b^	22.12 ± 0.94 ^c^	18.80 ± 0.84 ^c^	91.74 ± 3.63 ^a^	3.38 ± 0.10 ^e^	3.24 ± 0.66 ^e^	9.44 ± 0.06 ^d^	<0.001
Quercetin 3-glucoside	0.17 ± 0.02 ^cd^	0.25 ± 0.02 ^c^	0.48 ± 0.03 ^b^	0.41 ± 0.05 ^b^	0.39 ± 0.01 ^b^	2.33 ± 0.04 ^a^	0.04 ± 0.01 ^e^	0.06 ± 0.02 ^e^	0.11 ± 0.00 ^de^	<0.001
Sinapic acid	ND	ND	ND	ND	ND	ND	ND	ND	ND	-
Ellagic acid	ND	ND	ND	ND	ND	ND	ND	ND	ND	-
Phloridzin	ND	ND	ND	ND	ND	ND	ND	ND	ND	-
Myricetin	0.02 ± 0.00 ^bcd^	0.01 ± 0.00 ^d^	0.03 ± 0.01 ^bcd^	0.04 ± 0.00 ^bc^	0.04 ± 0.01 ^b^	0.09 ± 0.00 ^a^	<0.01 ± 0.00 ^d^	0.01 ± 0.00 ^d^	0.02 ± 0.01 ^cd^	<0.001
Resveratrol	ND	ND	ND	ND	ND	ND	ND	ND	ND	-
Pelargonidin	ND	ND	ND	ND	ND	ND	ND	ND	ND	-
Delphinidin	ND	ND	ND	ND	ND	ND	ND	ND	ND	-
Quercetin	0.04 ± 0.01 ^cd^	0.04 ± 0.00 ^cde^	0.07 ± 0.00 ^b^	0.03 ± 0.01 ^de^	0.05 ± 0.00 ^bcd^	1.35 ± 0.01 ^a^	0.01 ± 0.01 ^e^	0.01 ± 0.01 ^e^	0.06 ± 0.00 ^bc^	<0.001
Luteolin	2.42 ± 0.18 ^ef^	2.52 ± 0.06 ^ef^	5.70 ± 0.36 ^a^	4.91 ± 0.00 ^ab^	4.69 ± 0.19 ^bc^	3.92 ± 0.20 ^cd^	3.22 ± 0.23 ^de^	2.14 ± 0.36 ^f^	1.07 ± 0.01 ^g^	<0.001
Cyanidin	ND	ND	ND	ND	ND	ND	ND	ND	ND	-
Kaempferol	0.21 ± 0.02 ^abc^	0.08 ± 0.02 ^cde^	0.23 ± 0.06 ^a^	0.10 ± 0.06 ^bcde^	0.15 ± 0.00 ^abcd^	0.22 ± 0.01 ^ab^	0.06 ± 0.04 ^de^	0.01 ± 0.01 ^e^	0.03 ± 0.00 ^e^	<0.001
Naringenin	0.27 ± 0.01 ^c^	0.33 ± 0.01 ^b^	0.34 ± 0.01 ^b^	0.40 ± 0.00 ^b^	0.19 ± 0.01 ^a^	0.19 ± 0.00 ^d^	0.16 ± 0.00 ^de^	0.14 ± 0.00 ^e^	0.33 ± 0.01 ^b^	<0.001
Apigenin	0.45 ± 0.02 ^cde^	0.48 ± 0.01 ^cd^	0.68 ± 0.03 ^a^	0.50 ± 0.02 ^bc^	0.56 ± 0.03 ^b^	0.46 ± 0.01 ^cde^	0.41 ± 0.01 ^e^	0.24 ± 0.00 ^f^	0.42 ± 0.01 ^de^	<0.001
Sum of identified compounds	19.5 ± 0.4 ^d^	28.9 ± 0.3 ^c^	45.5 ± 2.1 ^b^	32.5 ± 1.0 ^c^	31.1 ± 0.9 ^c^	116.4 ± 4.2 ^a^	16.3 ± 0.4 ^de^	10.6 ± 1.3 ^e^	18.6 ± 0.1 ^d^	<0.001

ND = no data.

**Table 4:** In vitro analysis of adzuki bean leaves compared to lucerne hay (dry-weight basis). Entries in the same row with different superscript letters indicate significant differences based on a one-way ANOVA followed by post hoc Tukey testing at α = 0.05.

Analyte	Lucerne	AVTAB#5	AVTAB#6	AVTAB#7	AVTAB#8	AVTAB#9	ANOVA
Apparent dry matter digestibility (%)	62.18 ± 0.46	58.33 ± 2.55	58.95 ± 0.34	58.05 ± 1.59	63.13 ± 1.70	65.26 ± 3.92	0.066
Amylase neutral detergent fibre (%)	39.82 ± 1.40	40.93 ± 3.13	38.32 ± 3.45	41.66 ± 2.89	38.00 ± 3.13	39.78 ± 0.03	0.710
Ammonia (mg N NH_3_/L) ^1^	59.44 ± 15.83	51.73 ± 2.81	58.94 ± 11.61	45.26 ± 2.11	50.23 ± 2.81	77.09 ± 14.07	0.149
Total gas production (mL/g DM)	85.58 ± 5.77	73.84 ± 9.09	69.31 ± 10.06	72.99 ± 6.96	82.66 ± 10.05	73.26 ± 8.39	0.444
Methane production (mL/g DM)	9.49 ± 1.88	6.32 ± 1.6	6.69 ± 2.69	7.68 ± 1.11	6.33 ± 0.62	8.19 ± 1.04	0.418
Volatile fatty acid—Acetic (mM)	11.57 ± 1.10 ^a^	10.41 ± 0.51 ^ab^	9.40 ± 1.04 ^b^	10.32 ± 1.28 ^ab^	10.75 ± 0.47 ^ab^	10.10 ± 1.20 ^ab^	0.048
Volatile fatty acid—Propionic (mM)	4.04 ± 0.49 ^a^	3.10 ± 0.03 ^b^	2.74 ± 0.82 ^b^	3.31 ± 0.52 ^ab^	3.32 ± 0.03 ^ab^	3.54 ± 0.49 ^ab^	0.007
Volatile fatty acid—Butyric (mM)	4.49 ± 2.03	3.77 ± 0.44	4.09 ± 1.57	3.60 ± 1.84	4.45 ± 0.75	4.52 ± 1.32	0.805
Volatile fatty acid—Valeric (mM)	6.22 ± 1.32	4.77 ± 0.44	5.37 ± 0.92	4.80 ± 1.56	5.40 ± 0.32	5.76 ± 0.82	0.157

^1^ Average value for ammonia in blank was 275.99 ± 14.52 mg/dL.

**Table 5:** In vitro analysis of adzuki bean stems compared to lucerne hay (dry-weight basis). Entries in the same row with different superscript letters indicate significant differences based on a one-way ANOVA followed by post hoc Tukey testing at α = 0.05.

Analyte	Lucerne	AVTAB#5	AVTAB#6	AVTAB#7	AVTAB#9	ANOVA
Apparent dry matter digestibility (%)	62.18 ± 0.46 ^a^	56.52 ± 0.01 ^a^	57.06 ± 3.05 ^a^	58.66 ± 0.56 ^a^	47.59 ± 1.51 ^b^	0.002
Amylase neutral detergent fibre (%)	39.82 ± 1.40 ^c^	52.31 ± 0.41 ^b^	52.16 ± 0.53 ^b^	53.40 ± 1.86 ^b^	64.69 ± 0.57 ^a^	<0.001
Ammonia (mg N NH_3_/L) ^1^	59.44 ± 15.83 ^a^	20.64 ± 1.06 ^ab^	−1.99 ± 21.10 ^b^	26.86 ± 2.81 ^ab^	29.84 ± 3.52 ^ab^	0.03
Total gas production (mL/g DM)	85.58 ± 5.77	74.73 ± 5.87	60.42 ± 13.87	72.70 ± 4.32	58.54 ± 1.97	0.69
Methane production (mL/g DM)	9.49 ± 1.88	6.76 ± 1.75	5.65 ± 1.33	7.43 ± 0.53	4.71 ± 1.01	0.104
Volatile fatty acid—Acetic (mM)	11.57 ± 1.10 ^a^	9.06 ± 0.60 ^b^	9.17 ± 0.09 ^b^	9.33 ± 1.04 ^b^	7.39 ± 0.15 ^c^	<0.001
Volatile fatty acid—Propionic (mM)	4.04 ± 0.49 ^a^	3.72 ± 0.39 ^ab^	3.30 ± 0.10 ^b^	3.89 ± 0.54 ^ab^	3.92 ± 0.07 ^ab^	0.039
Volatile fatty acid—Butyric (mM)	4.49 ± 2.03	2.48 ± 1.22	2.67 ± 1.25	2.76 ± 0.97	2.36 ± 0.78	0.08
Volatile fatty acid—Valeric (mM)	6.22 ± 1.32 ^a^	2.10 ± 0.72 ^b^	2.21 ± 0.72 ^b^	2.42 ± 0.76 ^b^	2.07 ± 0.40 ^b^	<0.001

^1^ Average value for ammonia in blank was 275.99 ± 14.52 mg/dL.

## Data Availability

The data that support the findings of this study are available from the corresponding author upon reasonable request. The data are not publicly available to protect the intellectual property of the AgriVentis Technology Ltd. Australia.
